# Geometric and physical interpretation of the action principle

**DOI:** 10.1038/s41598-023-39145-y

**Published:** 2023-07-26

**Authors:** Gabriele Carcassi, Christine A. Aidala

**Affiliations:** Physics Department, University of Michigan, Ann Arbor, MI, 48109 USA

**Keywords:** Physics, Applied mathematics

## Abstract

We give a geometric interpretation for the principle of stationary action in classical Lagrangian particle mechanics. In a nutshell, the difference of the action along a path and its variation effectively “counts” the possible evolutions that “go through” the area enclosed. If the path corresponds to a possible evolution, all neighbouring evolutions will be parallel, making them tangent to the area enclosed by the path and its variation, thus yielding a stationary action. This treatment gives a full physical account of the geometry of both Hamiltonian and Lagrangian mechanics which is founded on three assumptions: determinism and reversible evolution, independence of the degrees of freedom and equivalence between kinematics and dynamics. The logical equivalence between the three assumptions and the principle of stationary action leads to a much cleaner conceptual understanding.

## Introduction

While the principle of stationary action is regarded by many as one of the most important tools in physics, its physical meaning is not completely clear^1–5^. First of all, the typical characterization of the Lagrangian as the difference between kinetic and potential energy fails even for simple systems, like a charged particle under a magnetic field. Moreover, the Lagrangian for a system is not uniquely defined, making the actual value of the action for a path not directly physically significant. We are left to wonder: what exactly is the action and why is it stationary for actual trajectories?

As part of our larger project Assumptions of Physics, we developed an approach, called Reverse Physics^6^, which examines current theories to find a set of starting physical assumptions that are sufficient to rederive them. We have found that Lagrangian mechanics is equivalent to three assumptions: determinism/reversibility, independence of degrees of freedom and kinematics/dynamics equivalence^7^. This physically motivated understanding of the classical theory can be used to characterize both the physics and the geometry underlying the principle of stationary action. What we find is that this arises as a general mathematical feature of divergence-free fields (and closed two-forms), which are the appropriate tools to describe a flow that conserves the number of states. The assumption of equivalence between kinematics and dynamics (i.e. we can reconstruct the dynamical state simply by looking at the trajectory) is what allows us to express the principle in the usual form. The argument can proceed in the reverse direction: assuming the principle of stationary action recovers a dynamical system that exhibits those three physical assumptions.

The mathematics needed to run the argument is well established^8–11^. The two aspects that have been sorely lacking, and that we provide, are (1) a clear geometric interpretation of the action principle and (2) a tight connection between the math and the physics it represents. [This has been the hardest problem to solve, as the modern mathematical concepts and notation have departed from physical intuition. We will therefore use, instead of the standard notation of differential geometry, an extended version of the one physicists use in general relativity.] To make the result accessible to the widest audience, we first cover the case of a single degree of freedom using standard vector calculus. We then proceed to the case of multiple independent degrees of freedom using tools from differential geometry. The main article will present all the key points needed to follow the argument and its physical and geometric meaning, leaving the mathematical details and calculations to the Supplementary Information.

## One degree of freedom

**Figure 1:**
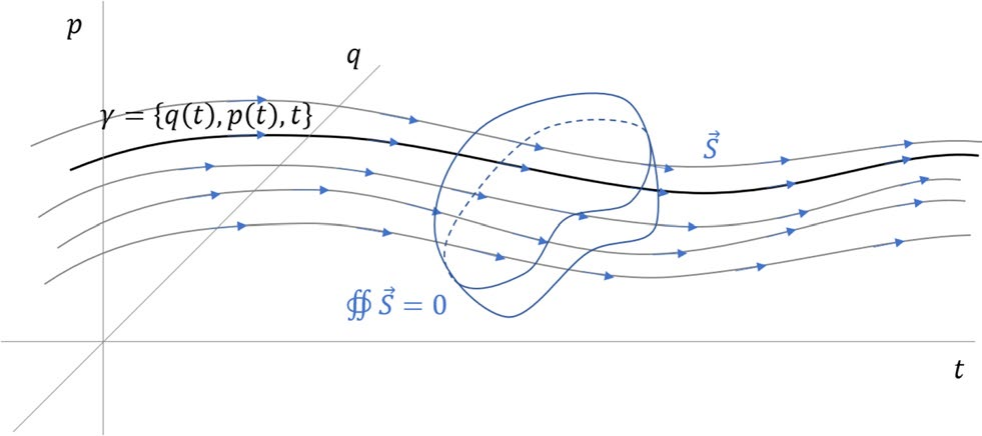
Evolutions in the extended phase space and the divergence-free displacement field.

As we want to characterize the evolution of states over time, the appropriate setting is phase space extended with the time variable^12,13^. That is, the space charted by position *q*, momentum *p* and time *t* as can be seen in Fig. 1. In the same way that we write $x^i = [ x, y, z ]$ for the three dimensions of space, we write

1 $$\begin{aligned} \xi ^a = [ q, p, t] \end{aligned}$$

for the three dimensions of the extended phase space.

Under the assumption that

DR $$\begin{aligned}&the\,\,system \,\,undergoes \,\,deterministic \,\,and \,\,reversible \\&evolution \end{aligned}$$

we can define a displacement vector field

2 $$\begin{aligned} \begin{aligned} \vec {S}&= \left[ \frac{dq}{dt},\frac{dp}{dt},\frac{dt}{dt} \right] \\&= S^a e_a = \frac{d\xi ^a}{dt} e_a. \end{aligned} \end{aligned}$$

that describes how states move in time. [Where possible, we will be writing the same expression in both vector calculus and component notations.] In dynamical system literature, this is referred to as the vector field of the dynamical system. The time component of the displacement vector field is constrained, as we have

3 $$\begin{aligned} S^t=\frac{dt}{dt}=1. \end{aligned}$$

If assumption (DR) is valid, we expect the flow of states through a closed surface to be zero: as many states flow in as flow out of the region. Alternatively, if we assign a probability, or probability density, to each trajectory, the assumption requires that probability not to change, so integrating the probability over a closed surface must yield zero. However we see it, assumption (DR) means the field is divergence-free. [Given that this is a three-dimensional space, we can use the standard tools of vector calculus.] That is,

4 $$\begin{aligned} \nabla \cdot \vec {S} = \partial _a S^a = 0. \end{aligned}$$

Since the displacement field is divergence-free, it admits a vector potential. We have

5 $$\begin{aligned} \begin{aligned} \vec {\theta }&= [\theta _q, \theta _p, \theta _t] = \theta _a e^a \\ \vec {S}&= - \nabla \times \vec {\theta } = - \epsilon ^{abc} \partial _b \theta _c \, e_a. \\ \end{aligned} \end{aligned}$$

The minus sign is introduced to match conventions. Mathematically, this is analogous to what is done for a magnetic field or for an incompressible fluid.

Because the displacement field must satisfy (3), without loss of generality we can set

6 $$\begin{aligned} \begin{aligned} \vec {\theta }&= [p, 0, -H(q,p,t)] \\&= p e^q - H(q,p,t) e^t, \end{aligned} \end{aligned}$$

where *H* is a suitable function of *q*, *p* and *t*. The potential $\vec {\theta }$ is closely related to the canonical one-form of symplectic geometry and the contact form of contact geometry. By applying definition (2) and expanding (5) with (6), we have

7 $$\begin{aligned} \left[ \frac{dq}{dt},\frac{dp}{dt},\frac{dt}{dt} \right] = - \nabla \times \vec {\theta } = \left[ \frac{\partial H}{\partial p},-\frac{\partial H}{\partial q}, 1 \right] , \end{aligned}$$

which yields Hamilton’s equations. Note that the argument works in reverse: any Hamiltonian system with one degree of freedom yields a divergence-free displacement field, and therefore satisfies (DR).

**Figure 2:**
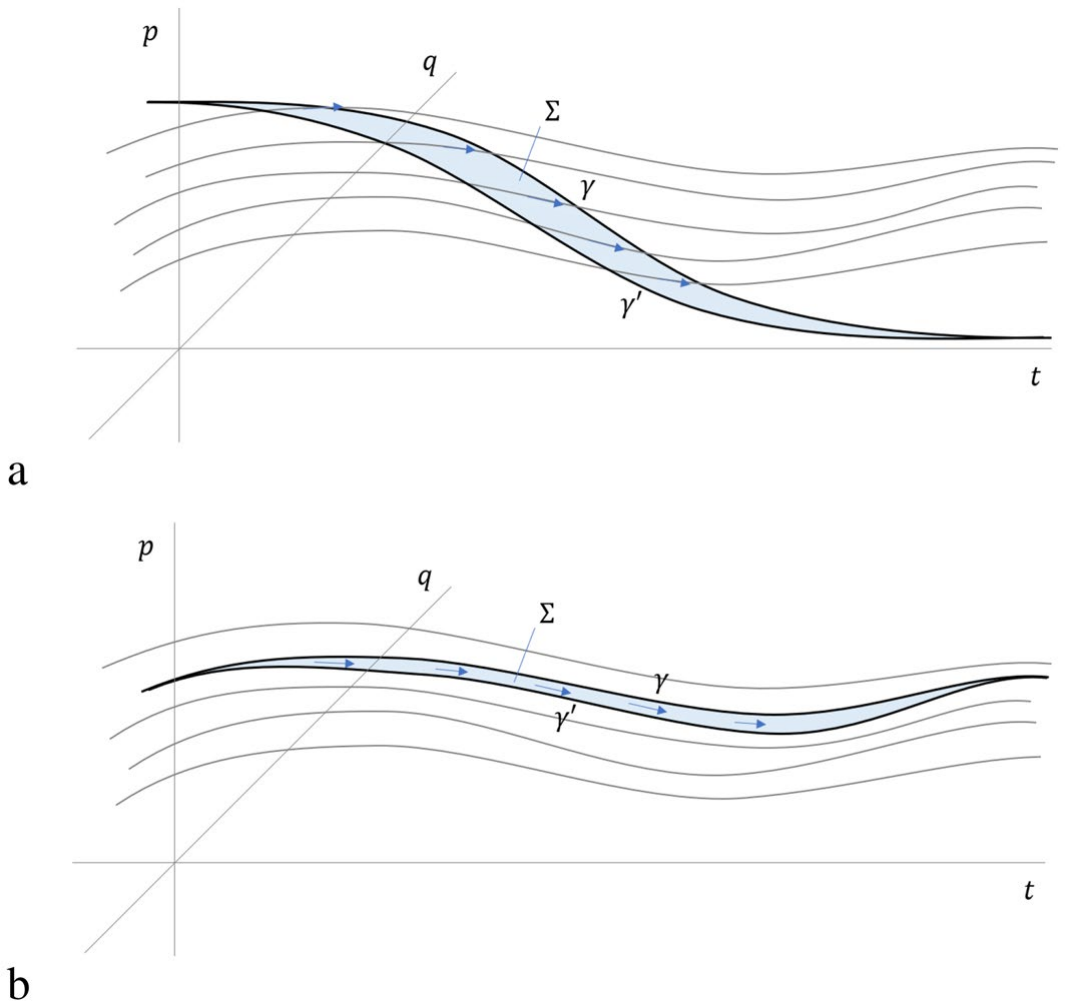
As shown in (**a**), the variation of the action is the flow of the displacement field $\vec {S}$ through the surface $\Sigma$ that sits between the path $\gamma$ and its variation $\gamma '$. In (**b**) we see that the flow is zero if the path is an actual evolution of the system, since the displacement field will be parallel to the path $\gamma$ and therefore tangent to the surface $\Sigma$.

We now turn to constructing the principle of stationary action. As illustrated in Fig. 2a, take a path $\gamma$ with endpoints *A* and *B*, not necessarily a solution of the equations of motion. Then take a variation $\gamma '$ of that path and identify a surface $\Sigma$ between them. We can ask: what is the flow of the displacement field $\vec {S}$ through $\Sigma$? Because $\vec {S}$ is divergence-free, the flow through $\Sigma$ will depend only on the contour, therefore the question is well posed. Using Stokes’ theorem, we find

8 $$\begin{aligned} \begin{aligned} - \iint _{\Sigma } \vec {S} \cdot d\vec {\Sigma }&= \iint _{\Sigma } \left( \nabla \times \vec {\theta } \right) \cdot d\vec {\Sigma } \\&= \oint _{\partial \Sigma = \gamma \cup \gamma '} \vec {\theta } \cdot d\vec {\gamma } \\&= \int _{\gamma } \vec {\theta } \cdot d\vec {\gamma } - \int _{\gamma '} \vec {\theta } \cdot d\vec {\gamma }' \\&= \delta \int _{\gamma } \vec {\theta } \cdot d\vec {\gamma }. \end{aligned} \end{aligned}$$

Now suppose $\gamma$ is a solution of the equation of motion, as in Fig. 2b. Then $\gamma$ is a field line and the flow is tangent to $\Sigma$ no matter what $\gamma '$ we picked. The converse is true: if we look for those paths for which the flow through $\Sigma$ is zero no matter what $\gamma '$, $\gamma$ must be everywhere tangent to $\vec {S}$ so we find a solution to the equation of motion. The solutions, then, are those paths and only those paths for which

9 $$\begin{aligned} 0 =\delta \int _{\gamma } \vec {\theta } \cdot d\vec {\gamma } = - \iint _{\Sigma } \vec {S} \cdot d\vec {\Sigma } \end{aligned}$$

We call this the principle of stationary action in Hamiltonian form.

The last step is to express the principle exclusively in terms of kinematic variables: position, time and velocity. This can be done if we assume that

KE $$\begin{aligned} the\,\, kinematics \,\,of \,\,the \,\,system \,\,is \,\,enough \,\,to \,\,reconstruct \,\,its \,\,dynamics. \end{aligned}$$

This means that by looking at just the trajectory in space *q*(*t*), we are able to reconstruct the state at each moment in time. Therefore we must be able to write $p=p(q,\dot{q})$, and therefore we can also write

10 $$\begin{aligned} \begin{aligned} \delta \int _{\gamma } \vec {\theta } \cdot d\vec {\gamma }&= \delta \int ^{t_2}_{t_1} \vec {\theta } \cdot \frac{d\vec {\gamma }}{dt} dt \\&= \delta \int ^{t_2}_{t_1} \left( p \frac{dq}{dt} - H \right) dt \\&= \delta \int ^{t_2}_{t_1}L(q, \dot{q}, t) dt = 0. \end{aligned} \end{aligned}$$

We find that a system for which (DR) and (KE) are valid can be characterized in terms of the principle of stationary action with a suitable Lagrangian. The converse is also true: if the principle of stationary action allows for a unique solution, then the conjugate momentum and the Hamiltonian are well defined and the system satisfies both (DR) and (KE).

We have thus demystified the principle of stationary action, and turned it into a geometric property: requiring the principle of stationary action is equivalent to requiring that the solutions are the field lines of a divergence-free field in phase space. We also have a clear physical meaning: the principle of stationary action is equivalent to assuming determinism/reversibility (DR) and kinematic equivalence (KE). However, we do feel that the principle expresses these requirements in a very roundabout way.

## Multiple degrees of freedom

To generalize to multiple degrees of freedom we have to abandon the tools provided by vector calculus and embrace the ones provided by differential geometry. For *N* independent degrees of freedom, we will have the $2N+1$ manifold charted by the variables

11 $$\begin{aligned} \xi ^a = [ q^i, p_i, t]. \end{aligned}$$

The displacement vector field will be

12 $$\begin{aligned} \begin{aligned} \vec {S}&= S^a e_a = \frac{d\xi ^a}{dt} e_a =\frac{dq^i}{dt} e_{q^i} + \frac{dp_i}{dt} e_{p_i} + \frac{dt}{dt} e_t \end{aligned} \end{aligned}$$

which also satisfies (3).

The flow of $\vec {S}$ will still be divergence-free, but this property only tells us that the total number of states is conserved. We also need to assume that

IND $$\begin{aligned} the \,\,degrees \,\,of \,\,freedom \,\,are \,\,independent. \end{aligned}$$

This means that, at each moment in time, the total number of states (i.e. the volume) of a parallelepiped will be the product of states identified by each degree of freedom (i.e. the areas). To be able to quantify the number of states identified by each degree of freedom, we introduce the rank-2 tensor (more precisely a two-form)

13 $$\begin{aligned} \omega = \omega _{ab} \, e^a \otimes e^b, \end{aligned}$$

which we call the state counting form. [Note that this approach is consistent with statistical mechanics^14^.]

The counting form must be of rank two as independent degrees of freedom are bi-dimensional (i.e. quantity plus conjugate). Given two vectors $\vec {v}$ and $\vec {w}$, these identify a parallelogram in phase space and

14 $$\begin{aligned} \omega (\vec {v}, \vec {w}) = \omega _{ab} v^a w^b \end{aligned}$$

quantifies the number of states over its surface.

The form $\omega$ will need to be anti-symmetric

15 $$\begin{aligned} \omega (\vec {v}, \vec {w}) = - \omega (\vec {w}, \vec {v}) \end{aligned}$$

as the parallelogram identified by $\vec {v}$ and $\vec {w}$ in that order will be the same as the one identified by $\vec {w}$ and $\vec {v}$ with opposite orientation. The form $\omega$ will also be closed, meaning16over all closed contractible surfaces. [In our notation, $d\Sigma$ represents the infinitesimal surface element of integration, which is the argument of the form.] This stems from (IND). Suppose you take a surface and translate it along another independent variable. The count of states cannot depend on the independent variable. Therefore if we construct any parallelepiped at equal time, two opposite sides will contain the same number of states with opposite orientation, and therefore the integral of $\omega$ over the whole surface will be zero. Those familiar with differential geometry will recognize $\omega$ as the symplectic form of symplectic manifolds and of the symplectic bundles of contact manifolds.

Since $\omega$ is closed, in every contractible region it can be expressed as the exterior derivative of a covector $\theta$:

17 $$\begin{aligned} \begin{aligned} \theta&= \theta _a e^a = \theta _{q^i} e^{q^i} + \theta _{p_i} e^{p_i} + \theta _t e^t \\ \omega&= - \partial \wedge \theta = - \left( \partial _a \theta _b - \partial _b \theta _a \right) e^a \otimes e^b \end{aligned} \end{aligned}$$

The minus sign is required to match conventions. [We write the exterior derivative as $\partial \wedge \omega$ instead of $d \omega$ so that *d* is only used for infinitesimal line or surface elements.]

The above is the generalization of (5) and it is important to note the differences. The exterior derivative takes the place of the curl and it is $\omega$, not $\vec {S}$, that can be expressed in terms of the potential $\theta$. Yet, there is an important geometric relationship we still need to take into account: the direction of the displacement does not contribute new states. Physically, this is a consequence of (DR): states cannot be created or disappear over time. We have

18 $$\begin{aligned} \omega (\vec {S}, \cdot ) = 0. \end{aligned}$$

Mathematically we say that the displacement vector kills the form; it makes it zero no matter what vector we put on the other side.

Similarly to the single d.o.f., without loss of generality we can set

19 $$\begin{aligned} \begin{aligned} \theta&= [p_i, 0, -H] \\&= p_i e^{q^i} - H e^t. \end{aligned} \end{aligned}$$

and calculate the components of the counting form

20 $$\begin{aligned} \omega _{ab} = (-\partial \wedge \theta )_{ab} = \begin{bmatrix} 0 &{}\quad \delta ^j_i &{}\quad \partial _{q^i} H \\ -\delta ^i_j &{}\quad 0 &{}\quad \partial _{p_i} H \\ -\partial _{q^j} H &{}\quad -\partial _{p_j} H &{}\quad 0 \end{bmatrix}. \end{aligned}$$

By combining (12), (18) and (20) one finds

21 $$\begin{aligned} \begin{aligned} S^{q^j}&= \partial _{p_j} H \\ S^{p_j}&= - \partial _{q^j} H \end{aligned} \end{aligned}$$

which recovers Hamilton’s equations.

The setup to recover the principle of stationary action is the same, except that we need to substitute vector calculus with differential geometry. First we find that the flow through $\Sigma$ is the variation of the action using Stokes’ theorem

22 $$\begin{aligned} - \iint _{\Sigma } \omega (d\Sigma ) = \delta \int _{\gamma } \theta (d\gamma ) \end{aligned}$$

If $\gamma$ is a solution of the equations of motion, then $d\gamma = \vec {S} dt$. The infinitesimal surface $d\Sigma$ will be a parallelogram formed by $d\gamma$ and the direction of the variation $d\lambda$. By (18), $\vec {S}$ is the only vector field that kills the form $\omega$ therefore the solutions are such that

23 $$\begin{aligned} 0 = \delta \int _{\gamma } \theta (d\gamma ) = - \iint _{\Sigma } \omega (\vec {S}, d\lambda ) dt. \end{aligned}$$

Adding assumption (KE), we can express the variational principle in the usual form

24 $$\begin{aligned} \begin{aligned} \delta \int _{\gamma } \theta (d\gamma )&= \delta \int ^{t_2}_{t_1}\left( p_i \frac{dq_i}{dt} - H \right) dt \\&= \delta \int ^{t_2}_{t_1}L(q^i, \dot{q}^i, t) dt = 0. \end{aligned} \end{aligned}$$

## Discussion and conclusions

We have found that the principle of stationary action is equivalent to assumptions (DR), (IND) and (KE) and that the variation of the action can be understood as the flow of evolutions through the surface delimited by the path and its variation. This allows us to fully understand both the physical and geometric significance of the principle in a much more precise way.

Note that the true physical content is in the displacement field $\vec {S}$ and the counting form $\omega$ whose properties descend from the assumptions. The action, the Lagrangian and the potential $\theta$ are not uniquely defined and are subject to a convenient, though still arbitrary, choice of gauge. The Lagrangian and the action, then, do not directly encode physical properties of the system. Saying that “nature chooses the path that minimizes the action”, therefore, does not provide clear insight. On the other hand, the three assumptions give us a clear physical picture.

Another interesting element is that a version of the principle of stationary actions holds even if (KE) does not apply. In this case the “Lagrangian” $L(q,\dot{q},p,t)$ depends on conjugate momentum as well. That is, the principle holds on all Hamiltonian systems, whether or not they admit a Lagrangian. Conversely, the cases where the Lagrangian does not admit a Hamiltonian are exactly the cases where the principle of stationary action fails to yield a unique solution, thus does not hold. These are the cases where the Lagrangian is not hyperregular. Therefore the principle of stationary action holds for all Lagrangian systems that admit a Hamiltonian, but also for all Hamiltonian systems whether of not they admit a Lagrangian. In this sense, the action principle is better understood as a feature of Hamiltonian mechanics, instead of Lagrangian mechanics^8,10^.

As a final comment, note that privileging the Hamiltonian picture is more in line with quantum mechanics; the idea of $\omega$ as a tool to count states is in line with statistical mechanics; expression (19) is remarkably similar to that of the relativistic four-momentum. This is one of the key insights we get from our Reverse Physics approach: there is a unity among the different physical theories that begs to be brought to light. We are convinced that a version of this geometric understanding must exist in the quantum world.

## Supplementary Information

Supplementary Information.

## Data Availability

All data generated or analysed during this study are included in this published article and its supplementary information files.
